# Missed case of Axenfeld-Rieger syndrome: a case report

**DOI:** 10.1186/1757-1626-1-299

**Published:** 2008-11-06

**Authors:** L Dhir, K Frimpong-Ansah, Nabil E Habib

**Affiliations:** 1 Royal Eye Infirmary, Apsley Road, Plymouth, PL4 6PL, UK

## Abstract

**Background:**

Anterior segment dysgenesis is a failure of normal development of the anterior segment of the eye. The structural anomalies are associated with glaucoma and corneal opacity which may lead to blindness.

**Case presentation:**

A Caucasian male was noted to have 'funny pupils' at the age of seven years but not followed up. He was diagnosed to have Axenfeld-Rieger syndrome at the age of thirty four years when he presented with glaucoma and visual field loss.

**Conclusion:**

Axenfeld-Rieger syndrome is uncommon. There is risk of sight loss due to glaucoma and corneal opacity. Importance of long-term follow up in cases of abnormal ocular findings in early life is emphasised.

## Background

Anterior segment dysgenesis is a failure of normal development of the anterior segment of the eye. The structural anomalies of the mature anterior segment are associated with increased risk of glaucoma [1,2]. We report a case noted to have 'funny pupils' at seven years of age but not followed up, until he presented to an optician with glaucoma at the age of thirty four years.

## Case presentation

A thirty four year old male Caucasian delivery driver presented to a High Street optician for the first time with a sore red eye. The optician diagnosed blepharitis and advised eyelid hygiene. Glasses were prescribed for a mild refractive error. High intraocular pressures were noted and a visual field defect was detected in the right eye. The optician observed pupillary abnormalities bilaterally, and hence referred the patient to the hospital for a definitive diagnosis.

Enquiry into past ophthalmic history revealed that the patient had been referred to the hospital at seven years of age as he had 'funny pupils' since birth. An ophthalmologist had noted congenital pupillary abnormalities, but had not arranged for further follow up. There was no past medical history or family history of note.

**Figure 1:**
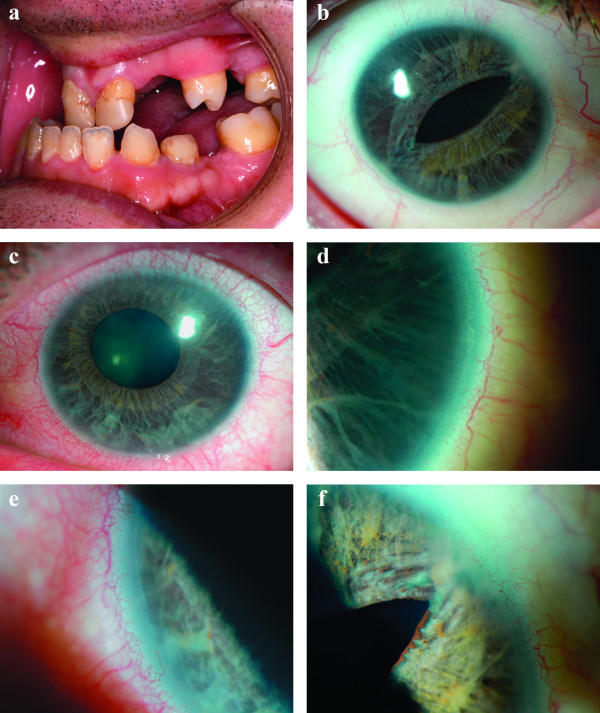
**1a **Microdontia and hypodontia. **1b **Slit pupil and iris atrophy right eye. **1c **Corectopia with iris atrophy left eye. **1d **Posterior embryotoxon right eye. **1e **Posterior embryotoxon left eye. **1f **Broad peripheral anterior synechiae right eye.

The patient had obvious maxillary hypoplasia, broad nasal bridge, telecanthus, microdontia and hypodontia. Only 23 adult teeth had erupted [Figure 1a]. Corrected visual acuities were 6/9 bilaterally. Pupillary abnormalities were markedly asymmetrical; right pupil was a mere slit, left was misshapen and ectopic. Iris atrophy was obvious [Figure 1b and 1c]. Slit lamp biomicroscopy and gonioscopy revealed posterior embryotoxon [Figure 1d and 1e], broad peripheral anterior synechiae to Schwalbe's line across 270° in the right angle [Figure 1f], but the left angle was open without peripheral anterior synechiae.

White to white corneal diameter was 10.5 mm in right eye and 10.75 mm in left. Left cornea showed superficial stromal opacities. Central corneal thickness was 636 and 589 microns and endothelial cell counts were 2537 and 2409 in right and left eye respectively. Intraocular pressures were 30 mmHg in right and 27 mmHg in left. On Humphrey's visual field (24-2) assessment, right eye showed infero-nasal step corresponding to supero-temporal notching of the optic disc. Left disc appeared healthy and the visual field was full.

A diagnosis of Axenfeld-Rieger syndrome was made. Anti-glaucoma drugs were prescribed and the intraocular pressure was successfully controlled with topical prostaglandin analogue and beta-blocker. The patient remains under close follow up at the Royal Eye Infirmary, Plymouth.

## Discussion

Anterior segment dysgenesis is a group of rare autosomal dominant conditions including posterior embryotoxon, Axenfeld-Rieger syndrome, Peter's anomaly and aniridia. Several different gene mutations, encoding for transcriptional regulators have been described. These specify migration and differentiation of mesenchymal progenital cells of neural crest origin into distinct anterior segment tissues. Interplay between PITX2 and FOXC1 explains phenotypic variability and genetic heterogeneity of anterior segment dysgenesis [1]. Main features are bilateral developmental ocular abnormalities which may be asymmetrical, umbilical cord anomalies, agenesis of certain teeth (usually maxillary incisors) and a hypoplastic mid-face. Alagille syndrome is associated with posterior embryotoxon in 95% of cases and is characterized by paucity of intrahepatic bile ducts, cardiopulmonary malformations and vertebral defects [3]. Posterior embryotoxon may be absent in Axenfeld-Rieger syndrome [4].

Ocular manifestations of Axenfeld-Rieger syndrome include iris stromal hypoplasia, ectropion uveae, corectopia, full-thickness iris defects, severe iris atrophy and extensive peripheral anterior synechiae. Glaucoma develops in 50% of cases, usually during early childhood or early adulthood, due to an associated angle anomaly or secondary synechial angle closure. Schlemm's canal may be small or absent, development of trabecular meshwork is aberrant and extracellular matrix is altered [2]. Elevation of intraocular pressure is initially managed medically, although surgery may be required subsequently.

## Conclusion

The ocular and systemic features of Axenfeld-Rieger syndrome are well-described in literature. The condition can lead to gradual and irreversible visual loss and needs specialist care and careful monitoring. Our case emphasises the grave importance of long-term follow up in cases of abnormal ocular findings noted at birth or early life, as these may only be a part manifestation of a potentially blinding syndrome, representing the proverbial tip of the iceberg.

## Competing interests

The authors declare that they have no competing interests.

## Authors' contributions

LD was involved in the care of the patient, drafting and writing the manuscript and literature review. NEH treated the patient and critically revised the manuscript. KF-A was involved with the treatment and investigations. All authors read and approved the final manuscript.

## Consent

Written informed consent was obtained from the patient for publication of this case report and accompanying images. A copy of the written consent is available for review by the Editor-in-Chief of this journal.
